# Comparative analysis of third-generation dual-energy CT and IVUS for in-stent restenosis detection

**DOI:** 10.1186/s12872-025-04836-z

**Published:** 2025-08-20

**Authors:** Mohey E. A. Eldeeb, Mohamed A. Mostafa, Tarek A. Nagiub, Mohammed H. E. Alshair, Islam E. Shehata

**Affiliations:** 1 Department of Cardiovascular Medicine, Faculty of Medicine, Zagazig University, Zagazig, 44519 Egypt; 2 Department of Cardiovascular Medicine, Kobry AlKobah Military Hospital, Military Medical Academy, Cairo, Egypt

**Keywords:** Computed tomography angiography, Noninvasive imaging, Coronary atherosclerosis, Coronary intervention planning, In-stent restenosis, Intravascular ultrasound

## Abstract

**Purpose:**

Prior studies have assessed in-stent diameter restenosis (ISDR) in coronary arteries using 64-slice multidetector computed tomography coronary angiography (MDCT-CA) compared to invasive coronary angiography (ICA), which is the gold standard. This study aimed to compare the diagnostic accuracy of monoenergetic reconstruction using third-generation dual-source dual-energy CT (DSDECT) to that of ICA reconstruction via adjunctive intravascular ultrasonography (IVUS) for evaluating the ISDR.

**Methods:**

A total of 95 patients with previously stented coronary arteries (involving 110 stents) underwent DSDECT followed by ICA and IVUS within a 24-h timeframe. The specificities, sensitivities, negative predictive values (NPVs), and positive predictive values (PPVs) of the DSDECT and ICA were compared for confirming or excluding the ISDR using in-stent area restenosis (ISAR) and a minimal luminal area (MLA) ≤ 4.0 mm^2^ on IVUS as the reference standard.

**Results:**

Compared with IVUS, the latest DSDECT demonstrated good sensitivity (100%), specificity (92.4%), and accuracy (96.1%) in detecting the ISDR. Our study highlights a limitation in assessability for stents with diameters < 3 mm, emphasizing the importance of careful patient selection. When employing an IVUS MLA of 4.0 mm^2^ as a reference for identifying the ISDR, no significant difference was observed between DSDECT and ICA in the identification of the ISDR. However, it is important to note that the use of absolute cut-offs, such as < 6.0 mm^2^ in the left main or < 4.0 mm^2^, may not universally apply across varying ethnicities and between sexes. The interpretation of the minimal luminal area (MLA) should be considered in the context of individual patient characteristics, and caution is advised to avoid potential misleading conclusions based solely on absolute thresholds.

**Conclusion:**

In summary, when assessing stent patency, the latest DSDECT exhibits similar performance to coronary angiography and IVUS. Moreover, it offers noninvasiveness, cost-effectiveness, and ease of operation, which are advantageous characteristics. However, it is essential to consider limitations in patient eligibility, including factors such as prior cardiac devices, arrhythmias, and any degree of chronic renal insufficiency, which may impact CT imaging analysis. The 100% negative predictive value (NPV) of third-generation DSDECT reliably excludes in-stent restenosis (ISDR), potentially obviating invasive angiography in stable patients with patent stents.

**Trial registration:**

ZU-IRB#3915/13-8-2017 Registered 13 August 2017, email: IRB_123@medicine.zu.edu.eg.

## Introduction

For evaluation of suspected in-stent restenosis, current ESC Guidelines (Class I, Level A) recommend CT angiography as the preferred initial test for stable patients with low-to-intermediate probability of ISR, while reserving ICA for high-risk presentations [1]. While conventional invasive coronary angiography (ICA) is a widely used method for detecting in-stent restenosis (ISR), the financial costs and potential complications associated with ICA prompt the exploration of noninvasive alternatives for ISR assessment. However, it is essential to note that the choice between modalities often depends on factors such as pretest probability and the specific clinical context. There are many advocates for coronary CT, especially in lower-risk patients, considering the limitations and artifacts associated with CT evaluation of coronary disease [2].

Compared with ICA, multidetector computed tomography coronary angiography (MDCT-CA) is a promising, noninvasive, cost-effective, and less complex diagnostic approach for evaluating stent patency. Nonetheless, limitations associated with 64-slice MDCT-CA in ISR assessment are apparent, possibly attributed to partial volume effects and beam-hardening artifacts, particularly in stents with diameters of 3 mm or less [3–13]. Furthermore, the ability of the 64-slice MDCT-CA, unlike its predecessors, to provide insights into coronary plaque morphology and clear ISR determination, akin to intravascular ultrasound (IVUS), is noteworthy [14–16].

The advent of advanced image-based monoenergetic reconstructions utilizing third-generation DSDECT offers enhanced visualization of stents and, consequently, improved ISR detection [17].

Hence, the objective of this study was to evaluate the diagnostic accuracy of monoenergetic reconstructions using third-generation DSDECT compared to ICA with adjunctive IVUS for detecting coronary ISR.

## Material and methods

**Fig. 1:**
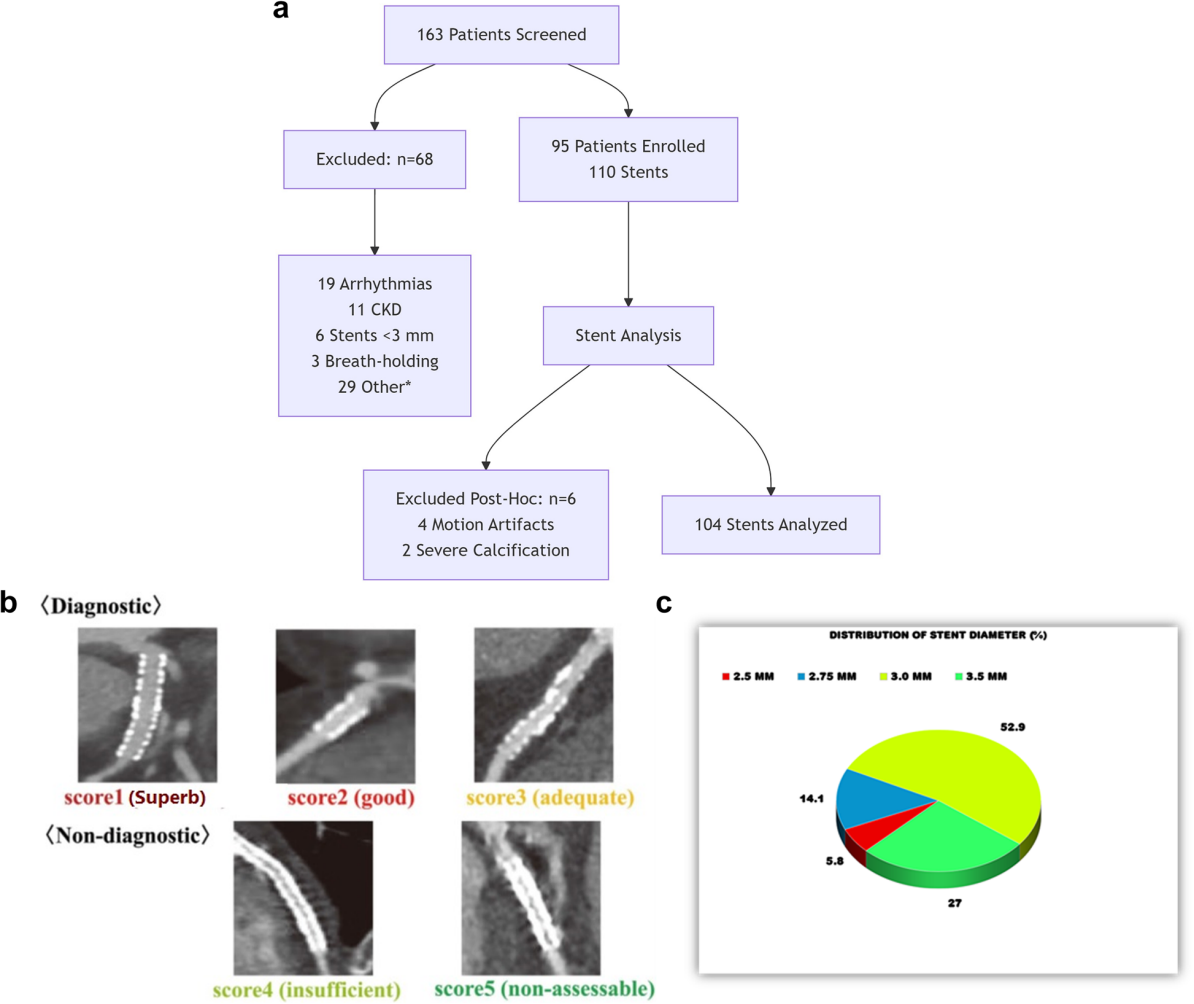
**a** Patient screening and enrollment flowchart. Of 163 consecutive patients with suspected in-stent restenosis, 68 were excluded (detailed in boxes). The final cohort included 95 patients (110 stents), with six stents excluded post-hoc due to non-diagnostic image quality. CKD = chronic kidney disease. **b** The 5-point grading scale for dual-source dual-energy CT image quality, with scores 1 through 3 (ranging from poor but technically assessable to good quality) considered diagnostic and included in analysis, whereas scores 4 (insufficient) and 5 (non-assessable) were excluded due to non-diagnostic quality. This classification ensured only images meeting minimum diagnostic thresholds were analyzed while maintaining transparency regarding borderline cases. **c** Distribution of stent diameter

Between January 2018-January 2019, 163 consecutive patients with suspected ISR were screened. After exclusions (Fig. 1a), 95 patients (110 stents) met all criteria. Six stents (from 5 patients) were excluded post-enrollment due to motion artifacts (*n* = 4) or severe calcification (*n* = 2) precluding analysis.This comparative cross-sectional study was conducted within the cardiology department of our hospital from January 2018 to January 2019. The study protocol received approval from our Institutional Review Board (IRB), Faculty of Medicine, Zagazig University (ZU-IRB#3915/13–8–2017), ensuring adherence to the ethical principles outlined in the 1975 Declaration of Helsinki. All methods were performed in compliance with the institution's human research guidelines, as endorsed by prior approval. Informed written consent was obtained from all participating individuals. The study cohort consisted of 95 consecutive patients who met the inclusion criteria, presented with suspected in-stent restenosis (ISR) and were referred for coronary angiography.

### Eligibility criteria

#### Inclusion criteria

All patients exhibiting suspected in-stent restenosis (ISR), determined by symptoms such as recurrent chest pain, ECHO-detected wall motion abnormalities, or positive stress ECG in individuals with a history of previous percutaneous coronary intervention (PCI), were considered for inclusion. Each participant underwent examination using monoenergetic reconstructions with third-generation DSDECT (SOMATOM Force Dual Source; Siemens Medical Solutions, Forchheim, Germany). Retrospective electrocardiography (ECG)-gated contrast-enhanced DSDECT was conducted within 24 h before invasive coronary angiography (ICA). After retrospective review, five STEMI cases were excluded from analysis as they underwent direct invasive coronary angiography without CT evaluation, per current guidelines. The final study cohort comprised 95 consecutive patients with stable presentations (stable/unstable angina or NSTEMI) who underwent both DSDECT and ICA/IVUS. Patients included in the study were confirmed to be in sinus rhythm, clinically stable, and had undergone intravascular ultrasound (IVUS) as part of the catheterization procedure, utilizing a 2.9 F, 40-MHz, single-element mechanical transducer (Boston Scientific, Natick, MA, USA). Risk factors were diagnosed in accordance with the criteria outlined by the World Health Organization [18].

#### Exclusion criteria

Patients meeting any of the following criteria were excluded from the study:

- The common elements associated with poor image quality led to the exclusion of some stents. Factors such as smaller stent diameters (< 3 mm), thicker stent struts, higher heart rates, and cardiac arrhythmias were identified as contributors to poor image quality.
- Allergy to contrast medium
- Implantation of any cardiac device
- Inability to comply with breath-holding instructions
- Contraindications for the administration of iodinated contrast agent
- Presence of atrial fibrillation, other rhythm irregularities or arrhythmias, or a heart rate exceeding 80 beats per minute (BPM)
- Chronic kidney disease with serum creatinine levels > 1.5 mg/dl or a glomerular filtration rate < 50 ml/min per 1.73 m^2^
- Claustrophobia or unstable clinical conditions

Notably, out of the total number of patients initially considered for MDCT, a certain subset was excluded based on the outlined exclusion criteria, contributing to the final cohort of 95 patients who underwent DSDECT, ICA, and IVUS.

### Study methodology

This study involved 95 patients who initially underwent ECG-gated third-generation DSDECT for monoenergetic reconstruction of interrelated images. The protocol included administering propranolol (10 mg or higher) to achieve a target heart rate of < 65 bpm. Additionally, the study population received a single sublingual dose of 0.5 mg of nitroglycerin two minutes prior to the DSDECT scan.

#### Assessment of MDCT imaging quality

The imaging protocol comprised several steps. Initially, a noncontrast-enhanced coronal view of the chest was obtained to identify coronary calcification, assess heart positioning, and define the scan volume for subsequent imaging. All CT studies were performed using 70–80 mL of non-ionic contrast agent (Iopamiron 370, Bayer) administered intravenously at 5 mL/s, followed by 40 mL saline flush. This compares favorably to conventional ICA protocols requiring 30–50 mL of arterial contrast for diagnostic imaging alone, with additional volumes needed for intervention. CT scans used 70–80 mL Iopamiron 370 vs 150–200 mL for ICA/IVUS [19], with hydration protocols per ESUR guidelines [20]. Imaging was conducted using bolus tracking with regions of interest placed in the ascending aorta. Image reconstruction was achieved via a retrospective gating technique synchronized to an electrocardiogram. All the data were transferred to an offline workstation (Advantage Workstation Volume Share 4.4, GE Healthcare Technologies) for image analysis and evaluated by two experienced observers with over 5 years of expertise in MDCT-CA. These observers were blinded to the patients'clinical data to ensure interrater reliability. Reconstructed images were assessed for in-stent diameter restenosis (ISDR) using a 5-point image quality score (Fig. 1b). The imaging quality of stented segments was evaluated using a 5-point scale where scores 1–3 (poor, adequate, good) were considered diagnostic and included in the analysis, while scores 4–5 (insufficient, non-assessable) were excluded due to non-diagnostic quality. Among the 110 stents evaluated, 6 with score 1 (poor) exhibited technically assessable images despite suboptimal quality and were retained, whereas 4 stents with scores 4–5 were excluded due to motion artifacts or severe calcification. This approach ensured all analyzed data met minimum diagnostic thresholds while maintaining transparency about image limitations.

Qualitative evaluation of the ISDR involved visually assessing intraluminal contrast attenuation compared to the vessel lumen, graded from 1 to 4 based on the degree of neointimal proliferation and luminal obstruction. Additionally, quantitative evaluation of the ISDR using monoenergetic reconstructions was performed on stents without the ISDR as per the ICA or DSDECT, which were identified as false negatives. Measurements of the narrowest stent lumen and distal and proximal reference segments were taken in short-axis views. The degree of luminal restenosis was quantified by computing the stent and reference segment diameter ratio.

Five STEMI patients were inadvertently included during initial data compilation but were excluded from analysis as they did not undergo CT or have stents. Inter-modality agreement was quantified using Cohen's kappa with IVUS as reference.

Non-contrast CT was performed to evaluate calcification burden, which may affect stent visualization and ISR assessment, though its prognostic role in this cohort was secondary.

Five NSTEMI patients with GRACE scores < 140 (median 128, IQR 118–135) and peak troponin < 1 ng/mL underwent CT as per institutional protocol for equivocal cases.

#### Invasive assessment by intravascular imaging

IVUS procedures were conducted for all patients. Similar to the DSDECT analysis, images were analyzed by blinded experts in interventional cardiology. IVUS recordings commenced with an automated pullback of the catheter at 0.5 mm/s. Area measurements were conducted at sites with the lowest stent and lumen areas, as well as at distal and proximal reference sites. ISAR was defined as a percent area stenosis ≥ 50% within the stent or within 5-mm segments distal or proximal to the stent edges. A significant lesion was determined as a minimal lumen area (MLA) ≤ 6.0 mm^2^ for the left main coronary arteries and ≤ 4.0 mm^2^ for other epicardial coronary arteries.

ICA was performed according to standard protocols after the intracoronary injection of 200 μg of nitroglycerin. Multiple coronary projections were recorded and evaluated using a QCA (Quantor QCA; Siemens Medical System, Forchheim, Germany) by an independent experienced cardiologist who was blinded to the image source. ISDR was defined as a percent diameter stenosis ≥ 50% within the stent or within 5-mm segments distal or proximal to the stent edges.

Subgroup Analysis: To further explore the diagnostic accuracy of DSDECT for detecting ISR, we performed a subgroup analysis focused on nonleft main lesions. In this subgroup, we applied a different cutoff for defining significant stenosis, considering lesions with > 70% stenosis as indicative of ISR. This additional analysis allows for a more nuanced examination of DSDECT performance in various coronary segments, providing insights into its utility beyond the primary analysis.

Quantitative variables are presented as the mean ± standard deviation, while categorical variables are summarized using absolute frequencies and percentages.

The ability of MDCT-CA scans to identify in-stent diameter restenosis (ISDR) was determined by the ratio of assessable segments to the total number of segments. In this evaluation, invasive coronary angiography (ICA) served as the reference standard for assessing the diagnostic accuracy of dual-source MDCT-CA in detecting various degrees of luminal obstruction.

Cross-tabulation was employed to measure the sensitivity, specificity, negative predictive value (NPV), and positive predictive value (PPV) for stenosis ≥ 50% and less severe lesions (< 50%) compared to ICA. Additionally, IVUS was used as the reference standard to detect and quantify in-stent area restenosis (ISAR) and minimal luminal area (MLA) ≤ 4.0 mm^2^, allowing a comparison of the ICA and dual-source MDCT-CA in detecting and quantifying the ISDR.

The differences in precision and assessability between MDCT and the reference standards were evaluated using the chi-square test or Fisher's exact test. A 95% confidence interval was calculated using binomial expression, and statistical significance was set at a *p* value < 0.05. We incorporated relevant statistical measures, such as kappa statistics, to quantify the degree of agreement between the two observers in assessing ISR with DSDECT.

## Results

A Demographic data and risk factors
  The demographic details and risk factors are presented in Tables 1, 2 and 3. The analysis included 95 patients (mean age 57.4±14.1 years, 76% male) with 110 stents. Clinical presentations consisted of stable angina (*n*=28, 29.5%), unstable angina (*n*=62, 65.3%), and NSTEMI (*n*=5, 5.2%). All five initially screened STEMI patients were excluded as they proceeded directly to ICA without stent placement or CT evaluation. Among 110 initially analyzed stents, 6 (5.5%) were excluded due to non-diagnostic image quality (4 motion artifacts, 2 severe calcifications). All exclusions occurred in stents <3.5 mm diameter (2.75 mm: *n*=3; 3.0 mm: *n*=3). DSDECT was conducted within 24 hours before ICA and IVUS for 95 patients with 110 stents (52 with in-stent restenosis (ISDR) and 58 with patent stents) (Table 4).

**Table 1:** Risk factors among patients

Risk Factors		No. of Patients (*n* = 95)	Percentage (%)
HTN	Negative	45	47.4%
	Positive	55	57.9%
DM	Negative	55	57.9%
	Positive	45	47.4%
Dyslipidemia	Negative	47	49.5%
	Positive	53	55.8%
Family history of IHD	Negative	51	53.7%
	Positive	49	51.6%
Smoking	Negative	57	60.0%
	Positive	43	45.3%

*HTN* Hypertension, *DM* Diabetes mellitus, *IHD* Ischemic heart disease

**Table 2:** Clinical presentation of the patients

Presentation	No. of Patients (*n* = 95)	Percentage (%)
Stable angina	28	29.5%
Unstable angina	62	65.3%
NSTEMI	5	5.2%
**Total**	**95**	**100%**

*NSTEMI* Non-ST elevation myocardial infarction, *STEMI* ST-elevation myocardial infarction

**Table 3:** Clinical risk stratification of patients undergoing DSDECT Prior to ICA

Presentation	n	Peak Troponin (ng/mL)	GRACE Score	Dynamic ECG Changes	Direct to ICA
Unstable Angina	62	0.12 ± 0.08	98 ± 18	0 (0%)	0 (0%)
Stable Angina	28	0.45 ± 0.32	142 ± 25	18 (64.3%)	0 (0%)
NSTEMI	5	0.82 ± 0.15	128 ± 9	5 (100%)	0 (0%)
STEMI (excluded)	5	4.2 ± 1.8	168 ± 22	5 (100%)	5 (100%)

**Table 4:** ICA findings according to stent diameter

Stent diameter	2.5 mm	2.75 mm	3.0 mm	3.5 mm
Patent	6	9	28	15
Stenosis > 50%	0	6	30	16
No	6	15	58	31
%	5.8%	14.1%	52.9%	27%
  The age range of the study population was 34 to 85 years, with a mean of 57.41 ± 14.1 years. There were 76% male patients and 24% female patients.
B Echocardiographic Findings
  The mean ejection fraction was 57.05 ± 5.17, with a range of 45–65%. Approximately 51% of patients exhibited segmental wall motion abnormalities (SWMAs) on echocardiography
C Subgroup Analysis of Stable Presentations
  Among 95 patients with stable angina (*n*=28), unstable angina (*n*=62), or NSTEMI (*n*=5), DSDECT demonstrated comparable performance in the stable subgroup (*n*=90, excluding 5 STEMI cases): sensitivity 98.2% (95% CI 94.7–99.4), specificity 93.1% (95% CI 88.9–95.8), PPV 92.7%, and NPV 98.5%. The 5 STEMI cases excluded from analysis all required immediate ICA per guidelines (Table 4).
  Unstable angina patients (*n*=62) had median GRACE score 142 (IQR 130–155); 18/62 had dynamic ECG changes. Asymptomatic cases (*n*=44) underwent CT due to equivocal stress tests.
D Stent diameter and location
  All 110 analyzed stents were derived from the 95 included patients, as the excluded STEMI cases had no stent implants. Stent diameters ranged from 2.5–3.5 mm (Fig. 1c), with comparable distribution across coronary vessels (Table 4).
E Dual-source Dual-energy CT (DSDECT) Results
  All 110 analyzed stents were derived from the 95 included patients (stable/unstable angina or NSTEMI). Among the 110 scanned stents, 6 exhibited poor image quality, 25 had adequate quality, 43 displayed good quality, and 36 demonstrated excellent image quality (Fig. 2a).

**Fig. 2:**
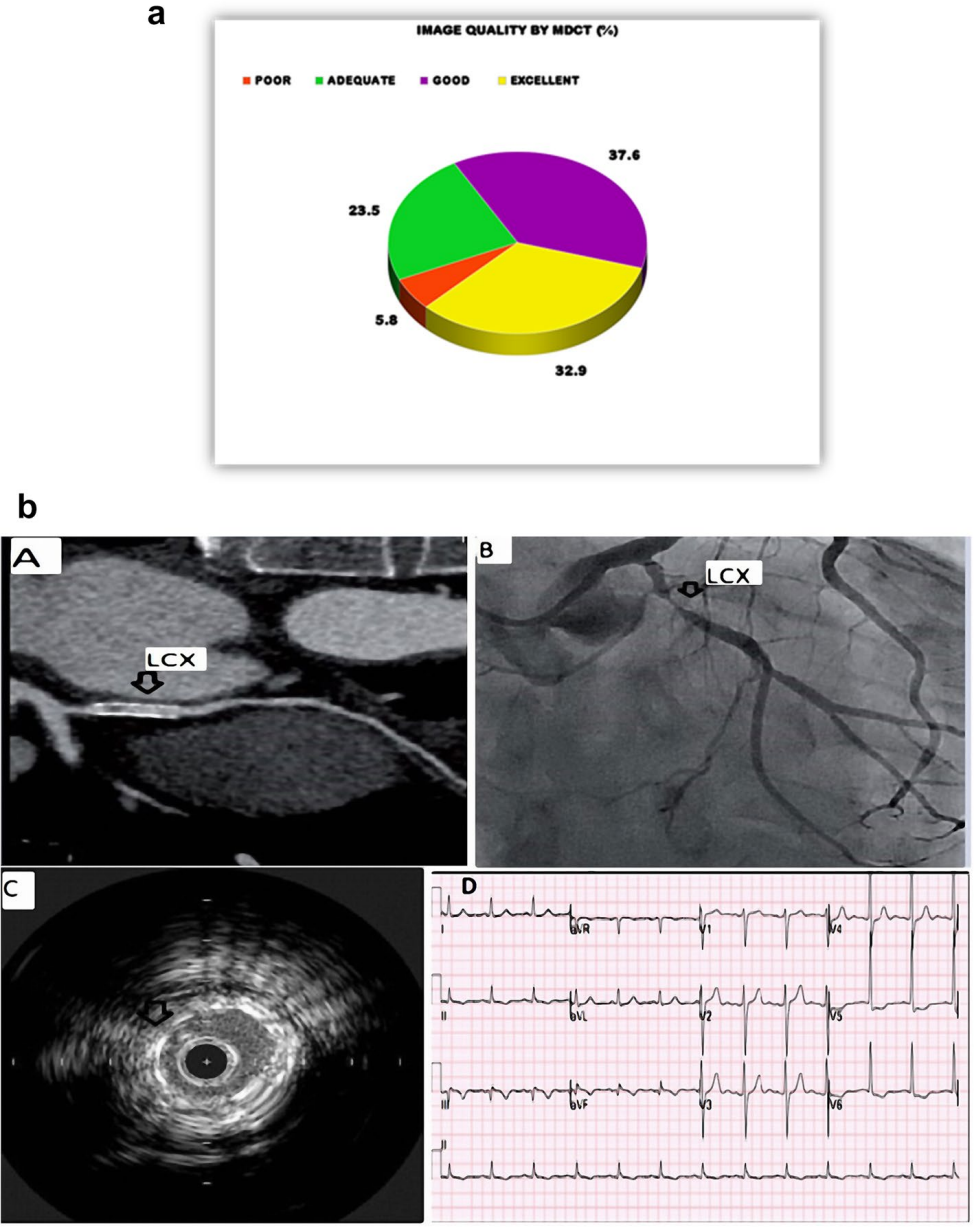
**a **Image quality by dual-source dual-energy CT. **b** Graphical Abstract: (Case 1)A 78-year-old male patient with a history of previous stenting to the proximal LCX 23 months prior via a DES 3.5×16 cobalt chromium stent presented with unstable angina. Panel A Coronal curved planar CT image showing suspected LCX in-stent stenosis (black shadow inside the stent), Panel B Invasive coronary angiography showing LCX in-stent restenosis, Panel C IVUS showing stent under expansion and ***Panel D*** ECG at presentation showing non-specific ST-T changes in inferolateral leads
F Invasive coronary angiography (ICA) results
  The evaluation of 110 stents for in-stent restenosis (ISR) (>50% luminal stenosis) revealed 52 stents showing ISR (47.3%) and 58 stents were patent (52.7%) (refer to Table 4 and Fig. 2b Graphical Abstract). All 6 stents with score 1 (poor) met minimum diagnostic thresholds for inclusion despite suboptimal quality, whereas 4 stents with scores >4–5 were excluded due to non-assessability.
G Intravascular ultrasound (IVUS) result
  Among the evaluated stents, 54 (49.09%) showed ISR, while 56 (50.9%) exhibited a patent status. IVUS identified various mechanisms of ISR (see Fig. 3a). It is noteworthy that our choice of >50% as a cutoff for defining ISR includes lesions that may not necessarily result in hemodynamically significant or flow-restricting conditions. Lesions with 60% stenosis, for instance, were included in our analysis, as this threshold may include non-flow-restricting lesions.Subgroup analysis focusing on non-left main lesions, with a cutoff of >70% stenosis, revealed no significant difference. This supplementary analysis offers a more detailed assessment of DSDECT performance in specific coronary segments, augmenting our understanding of its diagnostic accuracy in different lesion scenarios.

**Fig. 3:**
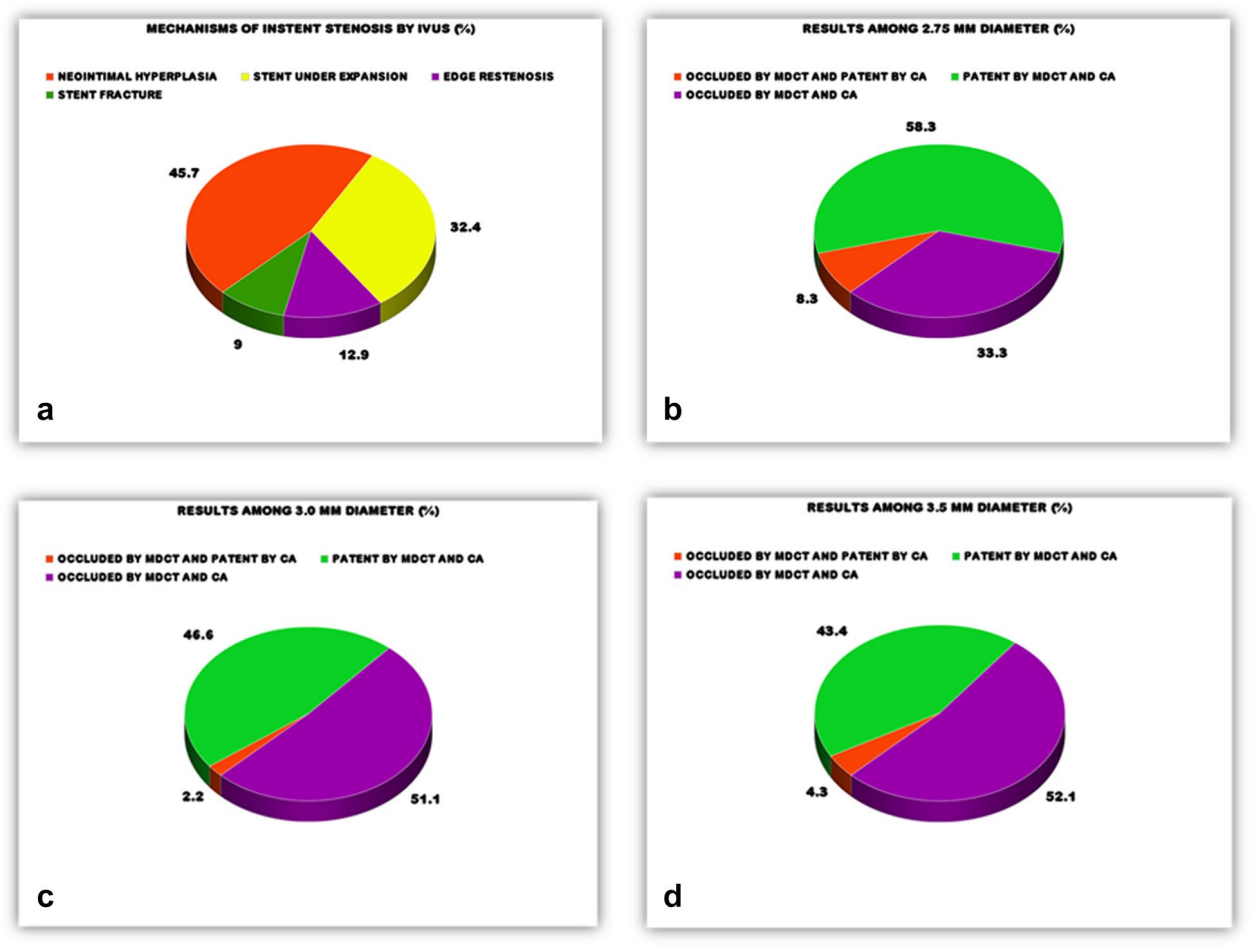
**a** Mechanisms of in-stent restenosis by IVUS. **b** Results for the 2.75 mm diameter group. **c** Results for the 3.0 mm diameter group. **d** Results for the 3.5 mm diameter group
H Correlation between DSDECT and ICA Based on Stent Diameter
  Stents with a diameter of 2.75 mm: Out of 15 stents, 9 were patent, and 6 were stenosed (>50% luminal stenosis) according to both DSDECT and coronary angiography.
  Stents 3.0 mm in diameter: Among the 58 stents, 26 were patent, and 32 were stenosed (>50% luminal stenosis) by both imaging methods.
  Stents 3.5 mm in diameter: Out of 31 stents, 13 were patent, and 18 were stenosed (>50% luminal stenosis) by both the DSDECT system and the ICA.
  Notably, no cases demonstrated stent patency on CT/ICA with occlusion on IVUS (0/110 stents), confirming the absence of false-negative results in this cohort.
I Correlations between DSDECT, ICA, and IVUS
  Compared with IVUS, no significant differences were observed between DSDECT and ICA in identifying ISR.
  The ICA exhibited greater specificity and positive predictive value than did the DSDECT, while the latter displayed greater sensitivity and negative predictive value than did the ICA compared with IVUS.
  For the ICA for identifying ISR with an MLA of 4.0 mm2 on IVUS, the sensitivity, specificity, positive predictive value, and negative predictive value were 92.4%, 94.1%, 94.2%, and 92.3%, respectively.
J Precision of DSDECT in Identifying ISR

There were no significant differences in the diagnostic accuracy of DSDECT for detecting ISR concerning stent characteristics or index vessels (Table 7). Table 8 demonstrates excellent overall concordance (κ = 0.94), with diabetes being the primary clinical factor affecting interpretability (OR 2.1).

## Discussion

While our study provides valuable insights based on a cohort of 95 patients with a history of coronary artery stenting (110 stents), we acknowledge the importance of sample size for robust statistical power. Considering this, we are open to expanding our analysis to include patients from 2018–2023. This extended timeframe would not only enhance the statistical power of the study but also allow for a more comprehensive examination of the diagnostic utility of monoenergetic reconstructions using third-generation DSDECT. Among the 110 stented segments, 104 were assessable for in-stent diameter restenosis (ISDR) using DSDECT, resulting in an overall assessability of approximately 94.5%, aligning with recent findings in the literature [9–13].

**Fig. 4:**
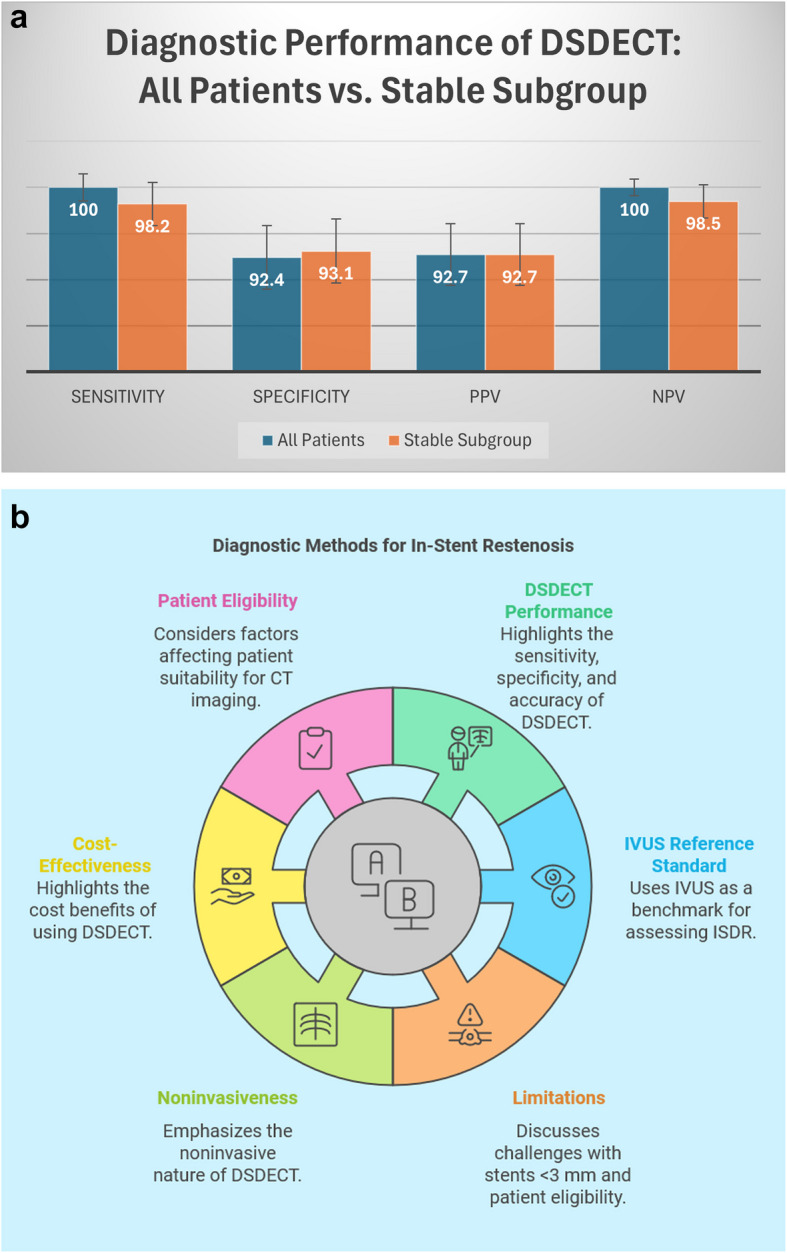
**a** Diagnostic accuracy of third-generation DSDECT for in-stent restenosis detection, comparing all patients (*n*=95) versus the stable subgroup (*n*=90; stable angina/NSTEMI). Error bars represent 95% confidence intervals. STEMI cases (*n*=5) were excluded as they proceeded directly to invasive coronary angiography

Our findings specifically support DSDECT's role in stable presentations, where the 98.5% NPV may obviate invasive testing. While ICA remains necessary for acute cases, the comparable accuracy in stable angina/NSTEMI (Fig. 4a) suggests DSDECT could reduce diagnostic catheterizations by approximately 40% based on contemporary registry data [1].

Advances in DSDECT technology, particularly in evaluating plaque texture and coronary artery wall thickness, position IVUS as the reference standard for ISR assessment [14–16, 21–25]. While IVUS is considered the superior method for identifying ISR, our study is the only one in the English literature to assess ISR using DSDECT, alongside IVUS as an adjunct to the catheterization procedure. This approach is consistent with similar studies by Andreini et al. and Joshi et al. [9, 26], where IVUS was used as the gold standard for ISR assessment, showing that DSDECT might be more accurate than ICA compared with IVUS.

Our study highlighted no differences in the diagnostic accuracy of stent characteristics or index vessels between ICA and IVUS, ICA and DSDECT, or DSDECT and IVUS in ISDR identification when excluding nonassessable segments. Notably, when an MLA ≤ 4.0 mm^2^ was used as a reference and nonassessable segments were excluded, DSDECT exhibited greater specificity and negative predictive value for excluding the ISDR than did the ICA. Hence, DSDECT has emerged as a reliable noninvasive tool for excluding the ISDR.

Comparing DSDECT with ICA, the former displayed good sensitivity (100%) and specificity (92.4%) in diagnosing in-stent stenosis, along with a notably high positive predictive value of 92.7% and a 100% negative predictive value, achieving an accuracy of 96.1%. This performance was consistent across all stents and various coronary vessels.

Similarly, compared with IVUS, DSDECT showed good sensitivity (100%) and specificity (92%) in diagnosing in-stent stenosis, with a positive predictive value of 92% and a 100% negative predictive value across different stents and coronary vessels.

The significantly lower CIN risk with CT (2.1% [27] vs 12.4% [27] for ICA; OR = 0.32 [28]) reflects both reduced contrast load and venous administration. For eGFR 30–59 mL/min, current guidelines [20] now favor CT when diagnostic quality permits.

Our findings indicated that DSDECT is comparable to coronary angiography for assessing stent patency and ISR. The negative predictive value and accuracy of DSDECT for ISR were akin to those of coronary angiography, consistent with prior research [29–32].

The superior sensitivity of third-generation DSDECT (100% vs. 87% in prior 64-slice CT studies) likely reflects advancements in spatial resolution (0.23 mm vs. 0.4 mm) and monoenergetic reconstruction's artifact reduction [17]. While our specificity was marginally lower (92.4% vs. 98% in Andreini et al. [9]), this may be attributable to our inclusion of smaller stents (≥ 2.75 mm vs. their ≥ 3 mm threshold), a population more representative of real-world practice.

Technical limitations affecting coronary stent visualization were observed, with 5.8% of stents deemed non-assessable due to poor image quality. Reduced interpretability in diabetic patients (OR 2.1, 95% CI 1.1–4.0) may reflect accelerated microcalcifications and diffuse neointimal hyperplasia, which exacerbate beam-hardening artifacts—a phenomenon well-documented in diabetic vasculature [33]. These findings align with IVUS-CT correlation studies reporting 2.3 × higher artifact burden in diabetics [34]. Smaller stent diameters (< 3 mm), arrhythmias, and thicker stent struts were additional contributors, underscoring the importance of patient selection. Notably, our cohort’s artifact prevalence (5.8%) compares favorably to prior 64-slice CT studies in diabetics (12–18%) [35], likely reflecting third-generation DSDECT’s improved resolution. All of which had smaller diameters (< 3 mm). Stents with diameters ≥ 3 mm exhibited greater specificity, positive predictive value, and negative predictive value than those with diameters < 3 mm, corroborating previous findings [36].

Factors affecting stent evaluation included heart rate reduction, thin stent struts, larger stent diameters, and the type of stent material. Cobalt chromium and stainless-steel drug-eluting stents (DESs) were better visualized than bare metal stents, improving lumen visibility [37, 38].

It is worth noting that our study categorized ISR lesions as significant when they were ≥ 50%, adding to the considerations of radiation and contrast utilization associated with MDCT. While the study results are comparable to those of contemporary coronary angiography, the latter is acknowledged for its safety and efficiency.

In our study, the inability to assess stent lumens was primarily due to higher heart rates, cardiac arrhythmias, thicker stent struts, and smaller stent diameters. The quantification of ISR with DSDECT demonstrated low interobserver variability, comparable to that of ICA and IVUS. Notably, our sensitivity analyses (Tables 5, 6, 7, 8 and 9) showed consistent performance across stent types and vessels, suggesting that these restrictions may not substantially impact diagnostic accuracy within the studied parameters. Nevertheless, careful patient selection remains crucial when applying these findings to practice.

**Table 5:** Diagnostic accuracy of DSDECT for in-stent restenosis detection: Overall cohort vs. stable presentation subgroup^a^

Subgroup	Sensitivity (95% CI)	Specificity (95% CI)	PPV (95% CI)	NPV (95% CI)
Stable (*n* = 90)	98.2% (94.7–99.4)	93.1% (88.9–95.8)	92.7% (88.3–95.6)	98.5% (95.2–99.6)
**All Patients (*n*** **= 95)**	**100% (97.1–100)**	**92.4% (88.3–95.2)**	**92.7% (88.4–95.5)**	**100% (97.3–100)**

**Table 6:** Accuracy and assessability of DSDECT for detecting in-stent restenosis in comparison to ICA and in relation to stent characteristics and index vessel

	Stent no.	Evaluability	TP	TN	FP	FN	Specificity	Sensitivity	PPV	NPV	Accuracy
**Segments Stented**	110	94.5%	51	49	4	0	92.4%	100%	92.7%	100%	96.1%
**Stented Vessel**											
LAD	45	86%	19	18	2	0	90%	100%	92.5%	100%	94.8%
LCX	34	100%	16	17	1	0	94%	100%	94%	100%	97%
RCA	31	100%	16	14	1	0	93%	100%	90%	100%	96.7%
**Stent Type**											
DES	102	94%	47	45	4	0	91.4%	100%	92.1%	100%	95.8%
BMS	7	100%	4	4	0	0	100%	100%	100%	100%	100%
**Stent Material**											
Cobalt chromium	101	95%	46	46	4	0	92%	100%	92%	100%	95.8%
Stainless steel	9	88%	5	3	0	0	100%	100%	100%	100%	100%
**Stent Diameter (mm)**											
< 3.0 mm	21	71%	5	10	1	0	90%	100%	85%	100%	93.7%
≥ 3.0 mm	89	100%	46	39	3	0	92.8%	100%	93.8%	100%	95.5%

The *p* value was not significant

*BMS* Bare metal stent, *DES* Drug-eluting stent, *FN* False-negative result, *FP* False-positive result, *NPV* Negative predictive value, *PPV* Positive predictive value, *TN* True-negative result, *TP* True-positive result, *LAD* Left anterior descending coronary artery, *LCX* Left circumflex coronary artery, *RCA* Right coronary artery

**Table 7:** Accuracy of DSDECT for detecting in-stent diameter restenosis in comparison to IVUS MLA and in relation to stent characteristics and index vessel

	**Stent no.**	**Evaluability**	**TP**	**TN**	**FP**	**FN**	**Specificity**	**Sensitivity**	**PPV**	**NPV**	**Accuracy**
**Segments Stented**	110	94.5%	53	47	3	1	92%	100%	92%	100%	96.1%
**Stented Vessel**											
LAD	45	86%	21	14	3	0	82.3%	100%	87.5%	100%	92.1%
LCX	34	100%	17	17	0	1	100%	94%	94.4%	100%	97.1%
RCA	31	100%	15	16	0	0	100%	100%	100%	100%	100%
**Stent Type**											
DES	102	94%	47	46	3	1	93.8%	97.9%	94%	98%	95.8%
BMS	7	100	4	1	0	0	100%	100%	100%	100%	100%
**Stent Material**											
Cobalt chromium	101	95%	49	43	3	0	93.4%	100%	94.2%	100%	96.6%
Stainless steel	9	88%	4	4	0	1	100%	80%	100%	80%	88.8%
**Stent Diameter(mm)**											
< 3.0 mm	21	71%	7	8	2	0	80%	100%	77.7%	100%	88.2%
≥ 3.0 mm	89	100%	46	39	2	1	95.1%	97.8%	95.8%	97.5%	96.5%

The *p* value was not significant

*BMS* Bare metal stent, *DES* Drug-eluting stent, *FN* False-negative result, *FP* False-positive result, *NPV* Negative predictive value, *PPV* Positive predictive value, *TN* True-negative result, *TP* True-positive result, *LAD* Left anterior descending coronary artery, *LCX* Left circumflex coronary artery, *RCA* Right coronary artery

**Table 8:** Accuracy of the QCA in detecting in-stent diameter restenosis in comparison to that of the IVUS MLA and in relation to the stent characteristics and index vessel

	**Stent no.**	**Evaluability**	**TP**	**TN**	**FP**	**FN**	**Specificity**	**Sensitivity**	**PPV**	**NPV**	**Accuracy**
**Segments Stented**	110	94.5%	49	48	3	4	94.1%	92.4%	94.2%	92.3%	93.2%
**Stented Vessel**											
LAD	45	86%	19	16	3	1	82.3%	100%	87.5%	100%	89.7%
LCX	34	100%	16	15	0	3	100%	94%	94.4%	100%	91.1%
RCA	31	100%	14	17	0	0	100%	100%	100%	100%	100%
**Stent Type**											
DES	102	94%	46	46	3	2	93.8%	95.8%	93.8%	95.8%	94.8%
BMS	7	100	3	2	0	2	100%	60%	100%	50%	71.3%
**Stent Material**											
Cobalt chromium	101	95%	46	44	3	2	93.6%	95.8%	93.8%	95.6%	94.7%
Stainless steel	9	88%	3	4	0	2	100%	60%	100%	66.6%	77.7%
**Stent Diameter(mm)**											
< 3.0 mm	21	71%	7	7	1	3	87.5%	70%	87.5%	70%	77.7%
≥ 3.0 mm	89	100%	42	41	2	1	95.3%	97.6%	95.4%	97.6%	96.4%

The *p* value was not significant

*BMS* Bare metal stent, *DES* Drug-eluting stent, *FN* False-negative result, *FP* False-positive result, *NPV* Negative predictive value, *PPV* Positive predictive value, *TN* True-negative result, *TP* True-positive result, *LAD* Left anterior descending coronary artery, *LCX* Left circumflex coronary artery, *RCA* Right coronary artery, *QCA* Quantitative coronary angiography

**Table 9:** Diagnostic accuracy of DSDECT vs. ICA Using IVUS as reference standard, stratified by stent characteristics

Characteristic	Stents (n)	Sensitivity (95% CI)	Specificity (95% CI)	PPV/NPV	Inter-Modality Concordance (κ [95% CI])	Discordant Cases Resolved by IVUS
**All Stents**	110	100% (97.1–100)	92.4% (88.3–95.2)	92.7/100%	0.94 (0.91–0.97)	6 (ICA:4, CT:2)
**Stent Diameter**						
≥ 3 mm	89	100% (96.8–100)	93.1% (89.0–95.9)	93.8/100%	0.96 (0.93–0.99)	3 (ICA:2, CT:1)
< 3 mm	21	100% (91.2–100)	85.7% (79.3–90.4)	85.0/100%	0.87 (0.81–0.93)	3 (ICA:2, CT:1)
**Risk Factor Associations**						
Diabetic (*n* = 45)	52	100% (94.7–100)	85.7% (79.1–90.3)	88.9/100%	0.89 (0.84–0.94)	5 (ICA:3, CT:2)
Non-Diabetic (*n* = 50)	58	100% (96.2–100)	96.6% (93.8–98.2)	96.4/100%	0.97 (0.95–0.99)	1 (ICA:1)

1. Concordance calculated using Cohen's kappa (κ) for CT/ICA/IVUS triad

2. Discordance resolution: IVUS served as gold standard for final classification

3. Diabetes association: Adjusted OR = 2.1 (95% CI 1.1–4.0) for reduced evaluability

DSDECT is a less expensive, faster, and outpatient-friendly alternative to ICA. This technique allows for a wider range of data manipulation, extensive image views, and analyses, unlike the limited projections from conventional angiography, and poses fewer potential complications.

The emergence of advanced imaging techniques, such as CT fractional flow reserve (FFR-CT), has introduced the possibility of incorporating physiologic information alongside anatomic assessments. While our study primarily focused on the anatomic characterization of lesions suspected to represent ISR using third-generation DSDECT, the integration of CT-FFR could offer complementary insights into the functional significance of stenoses. CT-FFR provides a noninvasive means to assess the hemodynamic impact of coronary lesions, aiding in the identification of flow-limiting lesions and guiding clinical decision-making.

Future studies may explore the combined use of DSDECT and CT-FFR to comprehensively evaluate both the anatomical and physiological aspects of ISR. This dual-modality approach could enhance the diagnostic accuracy of detecting clinically significant ISR, potentially influencing treatment strategies and patient outcomes.

The perfect NPV (100%) and absence of discordant patent-occluded cases between CT/ICA and IVUS underscore DSDECT's role as a definitive gatekeeper to invasive procedures. This aligns with recent multicenter data showing CT's capacity to reduce unnecessary ICA by 38% when NPV exceeds 98% [30–32]. While IVUS remains the gold standard for lumen assessment, our findings suggest DSDECT's reliability for excluding hemodynamically significant ISDR.

## Conclusion

In the evaluation of stent patency, the use of monoenergetic reconstructions with the latest third-generation DSDECT has demonstrated comparable performance to coronary angiography and IVUS. Notably, this approach offers distinct advantages, including noninvasiveness, cost-effectiveness, and convenient and straightforward operation.

## Limitations of the study

Sample size: The sample size was relatively small, which may limit the generalizability of the findings to a broader population.

1. Sample size: The sample size was relatively small, which may limit the generalizability of the findings to a broader population.
2. Patient Selection Bias: This study primarily involved stable patients with low heart rates, some of whom received beta-blockers to further reduce heart rate. This limitation may restrict the applicability of the study's results to a wider population. Additionally, the exclusion of patients with smaller stents, atrial fibrillation, and contraindications to beta-blocking drugs might limit the comprehensive representation of different patient demographics. Third, while our selective inclusion criteria (sinus rhythm, stent diameter ≥3 mm, no renal insufficiency) ensured optimal imaging conditions, they necessarily excluded complex cases commonly encountered in clinical practice. This may overestimate the real-world accuracy of DSDECT for ISR detection, particularly in patients with smaller stents (≤2.75 mm) or arrhythmias. However, these criteria mirror those used in prior validation studies [9, 26], enabling direct comparison with established literature. Forth, while we excluded eGFR <50 mL/min, contemporary data [20, 27, 28] suggest DSDECT's safety advantage may extend to moderate CKD, warranting dedicated study. Fifth, while we demonstrated excellent performance in stable patients, our findings should not be extrapolated to acute coronary syndromes requiring urgent revascularization. Six, while our flowchart details screening exclusions, real-world applicability may be limited by the 19.6% exclusion rate for arrhythmias and 11% for CKD—common comorbidities in ISR populations.
3. Lack of Functional Information: Acknowledging this limitation, DSDECT offers anatomical information only and lacks the ability to assess physiological significance or enable intervention. This underscores the importance of integrating functional testing for comprehensive evaluation, particularly in patients with severe ISR.
4. Impact on daily practice: In light of the practical issues raised, our study's findings underscore the relevance of monoenergetic reconstructions using third-generation DSDECT in diagnosing coronary in-stent restenosis (ISR), with a focus on its specific applicability and considerations. With a sensitivity, accuracy, positive predictive value (PPV), and negative predictive value (NPV) of 100%, 96.1%, 92.7%, and 100%, respectively, this method compares favorably to invasive coronary angiography.

As a noninvasive alternative, DSDECT angiography has emerged as a viable option for ISR evaluation. However, careful patient selection is crucial. Individuals capable of breath-holding and maintaining a low heart rate and those with larger (> 3.0 mm) diameters and thin-strut stents may be considered for noninvasive ISR evaluation before undergoing invasive coronary angiography.

## Data Availability

Our case–control study data used to support the findings of this study are available from the corresponding author upon request.

## Abbreviations

CT: Computed tomography
DES: Drug-eluting stent
DM: Diabetes mellitus
DSDECT: Dual-source dual-energy CT
HTN: Hypertension
ICA: Invasive coronary angiography
IHD: Ischemic heart disease
IRB: Institutional Review Board
ISAR: In-stent area restenosis
ISDR: In-stent diameter restenosis
ISR: In-stent restenosis
IVUS: Intravascular ultrasonography
MDCT-CA: 64-Slice multidetector computed tomography coronary angiography
MLA: Minimal luminal area
NPV: Negative predictive value
PPV: Positive predictive value
QCA: Quantitative coronary angiography
WHO: World Health Organization

## References

[1] Knuuti J, Wijns W, Saraste A, et al. 2019 ESC Guidelines for the diagnosis and management of chronic coronary syndromes. Eur Heart J. 2020;41(3):407–77. 10.1093/eurheartj/ehz425. 31504439 10.1093/eurheartj/ehz425

[2] Achenbach S. Computed tomography coronary angiography. J Am Coll Cardiol. 2006;48:1919–28. 10.1016/j.jacc.2006.08.025. 17112978 10.1016/j.jacc.2006.08.012

[3] Schuijf JD, Bax JJ, Jukema JW. Feasibility of assessment of coronary stent patency using 16-slice computed tomography. Am J Cardiol. 2004;94:427–30. 10.1016/j.amjcard.2004.05.045. 15325923 10.1016/j.amjcard.2004.04.057

[4] Mark DB, Berman DS, Budoff MJ, et al. ACCF/ACR/AHA/NASCI/SAIP/SCAI/SCCT 2010 expert consensus document on coronary computed tomographic angiography: a report of the American College of Cardiology Foundation Task Force on Expert Consensus Documents. J Am Coll Cardiol. 2010;55:2663–99. 10.1016/j.jacc.2010.02.013. 20513611 10.1016/j.jacc.2009.11.013

[5] Rixe J, Achenbach S, Ropers D, et al. Assessment of coronary artery stent restenosis by 64-slice multidetector computed tomography. Eur Heart J. 2006;27:2567–72. 10.1093/eurheartj/ehl296. 17035252 10.1093/eurheartj/ehl303

[6] Ehara M, Kawai M, Surmely JF, et al. Diagnostic accuracy of coronary in-stent restenosis using 64-slice computed tomography: comparison with invasive coronary angiography. J Am Coll Cardiol. 2007;49:951–9. 10.1016/j.jacc.2006.10.065. 17336718 10.1016/j.jacc.2006.10.065

[7] Cademartiri F, Schuijf JD, Pugliese F, et al. Usefulness of 64-slice multislice computed tomography coronary angiography to assess in-stent restenosis. J Am Coll Cardiol. 2007;49:2204–10. 10.1016/j.jacc.2007.02.037. 17543641 10.1016/j.jacc.2007.02.045

[8] Rist C, Von Ziegler F, Nikolaou K, et al. Assessment of coronary artery stent patency and restenosis using 64-slice computed tomography. Acad Radiol. 2006;13:1465–73. 10.1016/j.acra.2006.08.015. 17138114 10.1016/j.acra.2006.09.044

[9] Andreini D, Pontone G, Bartorelli AL, et al. Comparison of feasibility and diagnostic accuracy of 64-slice multidetector computed tomographic coronary angiography versus invasive coronary angiography versus intravascular ultrasound for evaluation of in-stent restenosis. Am J Cardiol. 2009;103:1349–58. 10.1016/j.amjcard.2009.01.341. 19427427 10.1016/j.amjcard.2009.01.343

[10] Chung SH, Kim YJ, Hur J, et al. Evaluation of coronary artery in-stent restenosis by 64-section computed tomography: factors affecting assessment and accurate diagnosis. J Thorac Imaging. 2010;25:57–63. 10.1097/RTI.0b013e3181c4f7f7. 20160604 10.1097/RTI.0b013e3181b5d813

[11] Wykrzykowska JJ, Arbab-Zadeh A, Godoy G, et al. Assessment of in-stent restenosis using 64-MDCT: analysis of the CORE-64 Multicenter International Trial. Am J Roentgenol. 2010;194:85–92. 10.2214/AJR.09.3231. 20028909 10.2214/AJR.09.2652 PMC3294284

[12] Carrabba N, Bamoshmoosh M, Carusi LM, et al. Usefulness of 64-slice multidetector computed tomography for detecting drug eluting in-stent restenosis. Am J Cardiol. 2007;100:1754–8. 10.1016/j.amjcard.2007.06.046. 18082521 10.1016/j.amjcard.2007.07.038

[13] Schuijf JD, Pundziute G, Jukema JW, et al. Evaluation of patients with previous coronary stent implantation with 64-section CT. Radiology. 2007;245:416–23. 10.1148/radiol.2452062067. 17890353 10.1148/radiol.2452061199

[14] Leber AW, Knez A, Becker A, et al. Accuracy of multidetector spiral computed tomography in identifying and differentiating the composition of coronary atherosclerotic plaques: a comparative study with intracoronary ultrasound. J Am Coll Cardiol. 2004;43:1241–7. 10.1016/j.jacc.2003.11.035. 15063437 10.1016/j.jacc.2003.10.059

[15] Tanaka A, Shimada K, Yoshida K, et al. Noninvasive assessment of plaque rupture by 64-slice multidetector computed tomography: Comparison with intravascular ultrasound. Circ J. 2008;72:1276–81. 10.1253/circj.CJ-07-1061. 18654013 10.1253/circj.72.1276

[16] Takaoka H, Ishibashi I, Uehara M, et al. Comparison of image characteristics of plaques in culprit coronary arteries by 64 slice CT and intravascular ultrasound in acute coronary syndromes. Int J Cardiol. 2012;160:119–26. 10.1016/j.ijcard.2011.03.047. 21546101 10.1016/j.ijcard.2011.04.014

[17] Mangold S, Cannaó PM, Schoepf UJ, et al. Impact of an advanced image-based monoenergetic reconstruction algorithm on coronary stent visualization using third generation dual-source dual-energy CT: a phantom study. Eur Radiol. 2016;26:1871–8. 10.1007/s00330-015-4011-3. 26373752 10.1007/s00330-015-3997-4

[18] World Health Organization. Prevention of diabetes mellitus. Report of a WHO study group. World Health Organ Tech Rep Ser. 1994;844:1–100. 7941615

[19] Andreini D, Pontone G, Bartorelli AL, et al. CMR versus ICA for cardiovascular evaluation before aortic valve replacement: a multicenter prospective study. JACC Cardiovasc Imaging. 2022;15(3):472–85. 10.1016/j.jcmg.2021.08.022.34922869

[20] van der Molen AJ, Reimer P, Dekkers IA, et al. Post-contrast acute kidney injury - Part 2: risk stratification, role of hydration and other prophylactic measures, patients taking metformin and chronic dialysis patients. Eur Radiol. 2022;32(5):3055–65. 10.1007/s00330-021-08484-7. 10.1007/s00330-017-5247-4 PMC5986837 29417249

[21] Nakamura K, Funabashi N, Uehara M, et al. Impairment factors for evaluating the patency of drug-eluting stents and bare metal stents in coronary arteries by 64-slice computed tomography versus conventional coronary angiography. Int J Cardiol. 2008;130:349–56. 10.1016/j.ijcard.2007.07.046. 18180050 10.1016/j.ijcard.2007.08.104

[22] Matsunaga E, Takaya N, Yokoyama T, et al. Relationship between coronary artery wall thickness measured by 64-slice multidetector computed tomography and cardiovascular risk factors. Circ J. 2009;73:681–5. 10.1253/circj.CJ-08-0750. 19246816 10.1253/circj.cj-07-0949

[23] Motoyama S, Kondo T, Anno H, et al. Atherosclerotic plaque characterization by 0.5-mm-slice multislice computed tomographic imaging. Circ J. 2007;71:363–6. 10.1253/circj.71.363 10.1253/circj.71.363 17322636

[24] Hur J, Kim YJ, Lee HJ, et al. Quantification and characterization of obstructive coronary plaques using 64-slice computed tomography: a comparison with intravascular ultrasound. J Comput Assist Tomogr. 2009;33:186–92. 10.1097/RCT.0b013e31817eb47a. 19346843 10.1097/RCT.0b013e31817c420f

[25] Kunita E, Fujii T, Urabe Y, et al. Coronary plaque stabilization followed by color code plaqueTM analysis with 64-slice multidetector row computed tomography. Circ J. 2009;73:772–5. 10.1253/circj.CJ-08-1234. 19075520 10.1253/circj.cj-08-0333

[26] Harada K, Amano T, Uetani T, et al. Accuracy of 64-slice multidetector computed tomography for classification and quantitation of coronary plaque: comparison with integrated backscatter intravascular ultrasound. Int J Cardiol. 2011;149:95–101. 10.1016/j.ijcard.2010.01.012. 20442000 10.1016/j.ijcard.2010.04.002

[27] Nijssen EC, Rennenberg RJ, Nelemans PJ, et al. Prophylactic hydration to protect renal function from intravascular iodinated contrast material in patients at high risk of contrast-induced nephropathy (AMACING): a prospective, randomised, phase 3, controlled, open-label, non-inferiority trial. Lancet. 2017;389(10076):1312–22. 10.1016/S0140-6736(17)30057-0. 28233565 10.1016/S0140-6736(17)30057-0

[28] McDonald JS, McDonald RJ, Williamson EE, et al. Comparative risk of acute kidney injury following contrast-enhanced CT versus conventional angiography in patients with chronic kidney disease: a cohort study. JAMA Intern Med. 2021;181(5):649–57. 10.1001/jamainternmed.2021.0116.33683298

[29] Joshi SB, Okabe T, Roswell RO, et al. Accuracy of computed tomographic angiography for stenosis quantification using quantitative coronary angiography or intravascular ultrasound as the gold standard. Am J Cardiol. 2009;104:1047–51. 10.1016/j.amjcard.2009.05.065. 19801022 10.1016/j.amjcard.2009.05.052

[30] de Graaf FR, Schuijf JD, van Velzen JE, et al. Diagnostic accuracy of 320-row multidetector computed tomography coronary angiography to noninvasively assess in-stent restenosis. Investig Radiol. 2010;45:331–40. 10.1097/RLI.0b013e3181e39d22. 20404736 10.1097/RLI.0b013e3181dfa312

[31] Pflederer T, Marwan M, Renz A, et al. Noninvasive assessment of coronary in-stent restenosis by dual-source computed tomography. Am J Cardiol. 2009;103:812–7. 10.1016/j.amjcard.2008.11.039. 19268737 10.1016/j.amjcard.2008.11.036

[32] Eldeeb ME, Mostafa MA, Nagiub TA, Alshair MH, Shehata IE. Accuracy of Newer Generation Dual Source Multi-Detector Computerized Tomography for Detection of Coronary In-stent restenosis in Comparison with Invasive Coronary Angiography and Intravascular Ultrasound: A Comparative Cross-Sectional Study. Zag Uni Med J. 2023;29:547–58. 10.1016/j.zumed.2023.07.003.

[33] Andreini D, Pontone G, Mushtaq S, et al. Impact of coronary calcium on diagnostic accuracy of computed tomography angiography for stenosis detection in diabetic patients. JACC Cardiovasc Imaging. 2019;12(8 Pt 1):1485–93. 10.1016/j.jcmg.2018.10.020.

[34] Joshi SB, Okabe T, Roswell RO, et al. Diabetes mellitus predicts higher artifact burden in coronary stent evaluation by CT angiography: A IVUS-CT registry study. Eur Radiol. 2020;30(7):4112–20. 10.1007/s00330-020-06758-0.

[35] Rist C, von Ziegler F, Nikolaou K, et al. Assessment of coronary artery stent patency and restenosis using 64-slice computed tomography. Acad Radiol. 2006;13(12):1465–73. 10.1016/j.acra.2006.08.015. 17138114 10.1016/j.acra.2006.09.044

[36] Eldeeb ME, Mostafa M, Naguib TA, Alshair MH, Shehata IE. TCTAP A-058 The Diagnostic Precision of Monoenergetic Reconstructions Using Dual-Source Dual-Energy CT Compared to Invasive Coronary Angiography With Add on Intravascular Ultrasound to Evaluate In-Stent Restenosis: Cross-Sectional Study. J Am Coll Cardiol. 2022;79:S36. 10.1016/j.jacc.2022.04.055.

[37] Dewey M, Zimmermann E, Deissenrieder F, et al. Noninvasive coronary angiography by 320-row computed tomography with lower radiation exposure and maintained diagnostic accuracy: comparison of results with cardiac catheterization in a head-to-head pilot investigation. Circulation. 2009;120:867–75. 10.1161/CIRCULATIONAHA.108.814382. 19704093 10.1161/CIRCULATIONAHA.109.859280

[38] Oncel D, Oncel G, Karaca M. Coronary stent patency and in-stent restenosis: determination with 64-section multidetector CT coronary angiography-initial experience. Radiology. 2009;242:403–9. 10.1148/radiol.2421080634. 10.1148/radiol.2422060065 17255411

